# A preliminary study on the prognostic significance of cysteine-rich EGF ligand domain 2 protein (CRELD2) in patients with triple negative breast cancer

**DOI:** 10.17305/bb.2024.11865

**Published:** 2025-01-24

**Authors:** Mehmet Zahid Kocak, Murat Araz, Siddika Findik, Aykut Demirkiran, Mustafa Korkmaz, Melek Karakurt Eryilmaz, Mehmet Artac

**Affiliations:** 1 Necmettin Erbakan University, Meram Faculty of Medicine, Department of Medical Oncology, Konya, Türkey; 2 Necmettin Erbakan University, Meram Faculty of Medicine, Department of Medical Pathology, Konya, Türkey

**Keywords:** Cysteine-rich epidermal growth factor ligand domain 2 protein, CRELD2, breast cancer, triple negative, survival

## Abstract

The cysteine-rich epidermal growth factor ligand domain 2 protein (CRELD2) is associated with pathways that regulate epithelial-to-mesenchymal transition, a critical process driving cancer metastasis. This study aimed to determine the prognostic value of CRELD2 status on survival outcomes in triple-negative breast cancer (TNBC). Seventy patients were included in the study. Thirty-four patients were metastatic, and 36 patients were non-metastatic. CRELD2 protein expression in tumor tissue was determined by immunohistochemical staining (IHC). The patients were divided into two groups: CRELD2 positive and negative groups. Clinicopathological features and survival outcomes were compared between the groups. In the survival analysis of the non-metastatic patient group, five-year overall survival (OS) rate was 91.7% in the CRELD2-positive patient group and 91% in the negative group (*P* ═ 0.91). Median progression free survival (PFS) was 9.4 (95% confidence interval [CI]: 6.4–12.4) months in the CRELD2-positive group and 11.9 (95% CI: 8.2–18.6) months in the CRELD2-negative group (*P* ═ 0.04). The median OS was 17.2 (95% CI: 13.7–22.3) months in the CRELD2-positive group and 24.7 (95% CI: 21.8–29.6) months in the CRELD2-negative group (*P* ═ 0.02). In multivariate analysis, CRELD2 status (negative vs positive) (hazard ratio [HR]: 0.50, 95% CI: 0.38–0.96, *P* ═ 0.02) was determined to be a risk factor for OS and CRELD2 status (negative vs positive) (HR: 0.82, 95% CI: 0.33–0.96, *P* ═ 0.01) was defined as a risk factor for PFS in patients with metastatic TNBC. This is the first clinical study to determine the effect of CRELD2 on survival and as a prognostic marker in patients with triple metastatic breast cancer. These results need to be validated prospectively with a large sample size.

## Introduction

The endoplasmic reticulum (ER) is an essential organelle responsible for folding and modifying newly synthesized proteins [1]. Under certain pathophysiological conditions, unfolded proteins can accumulate, impairing ER function [2]. This dysfunction caused by unfolded protein deposits is termed ER stress. Numerous genes induced by ER stress have been identified, with their expression regulating stress sensors—such as PERK, IRE1, and ATF6—that mediate the stress response [3, 4]. Among these, cysteine-rich epidermal growth factor ligand domain 2 protein (CRELD2) has been defined as an ER stress-inducible gene [5]. CRELD2 is a glycoprotein primarily localized in the ER and Golgi apparatus [6]. It plays intracellular and extracellular roles in both physiological and pathological contexts, though its molecular properties remain incompletely understood [6].

**Figure 1.**
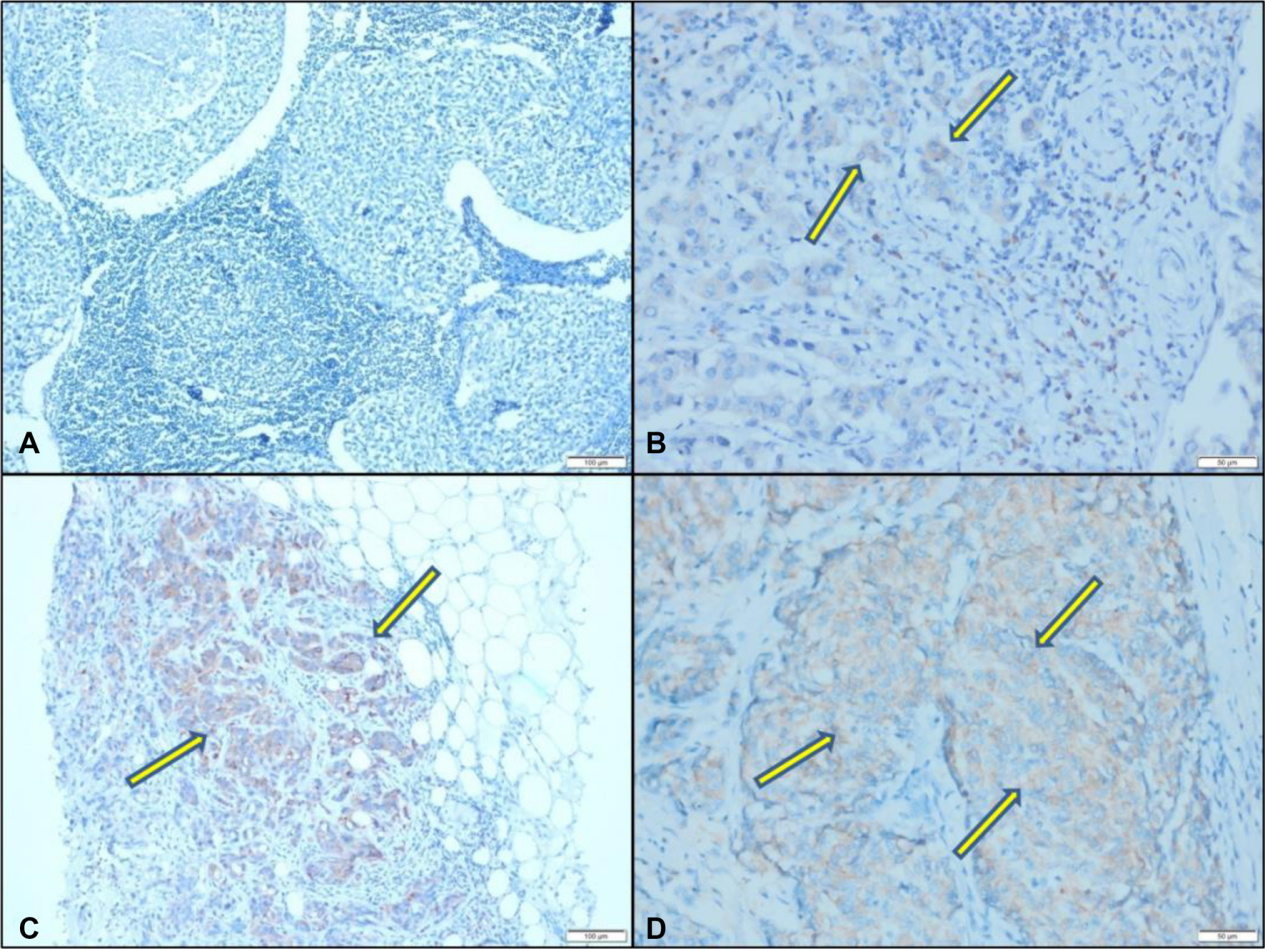
**Immunohistochemical staining of CRELD2 protein.** (A) Negative expression of CRELD2 (no staining) (×100 magnification); (B) CRELD2 expression in 1%–10% of tumor cells (×200 magnification); (C) CRELD2 expression in 11%–50% of tumor cells (×100 magnification); (D) CRELD2 expression in >50% of tumor cells (×200 magnification). CRELD2: Cysteine-rich epidermal growth factor ligand domain 2.

The molecular mechanisms governing cancer–microenvironment interactions are not yet fully elucidated. Cancer-associated fibroblasts (CAFs), key components of the cancer microenvironment, create an inflammatory environment and alter the biochemical properties of the extracellular matrix [7]. These CAF-mediated changes enhance the proliferation, survival, and metastasis of tumor cells across various cancer types [8–12]. Recent findings using a mouse model revealed that stress sensors increase CRELD2 protein synthesis, promoting tumor growth and proliferation by amplifying CAF activity in breast cancer—particularly in the triple-negative subtype [13]. The same study demonstrated higher CRELD2 expression levels in invasive breast carcinomas compared to normal tissue and showed that tumor progression was arrested when CRELD2 levels were reduced [13]. Additionally, CRELD2 has been implicated in pathways regulating epithelial-to-mesenchymal transition (EMT), a key process driving cancer metastasis [14]. Its involvement in ER stress signaling further suggests a potential role in tumor adaptation to hypoxia and nutrient deprivation, both hallmark features of the triple-negative breast cancer (TNBC) microenvironment [15].

Accounting for approximately 10%–20% of all breast cancer cases, TNBC disproportionately affects younger women and is associated with poor prognosis, high rates of metastasis, and limited therapeutic options. Unlike hormone receptor-positive or HER2-positive breast cancers, TNBC lacks targeted therapies and endocrine treatments, leaving chemotherapy as the primary treatment modality [16]. The urgent need for novel therapeutic targets and prognostic biomarkers has spurred research into TNBC’s molecular underpinnings. Recent studies have identified programmed cell death protein-1 (PD-L1) levels as predictive markers in TNBC patients [17, 18]. However, no clinical studies to date have evaluated the prognostic significance of CRELD2 in TNBC. It remains unclear whether CRELD2 positivity or expression levels are associated with survival outcomes. This study aimed to investigate the prognostic value of CRELD2 expression for survival outcomes in patients with TNBC.

## Materials and methods

### Patients’ population

Adults (>18 years) histopathologically diagnosed with TNBC (estrogen and progesterone receptor levels < 1%, and CerbB2 (Her-2) score 0–1 or score 2 with CISH/FISH-negative results) were retrospectively included in this study. Patients were treated in our medical oncology department between January 2010 and January 2019. Subtypes included one case of metaplastic carcinoma, one case of adenoid cystic carcinoma, and all others were invasive ductal carcinoma.

Although written consent was not obtained, ethics committee approval was granted by the local ethics committee (approval number: 2020/2892).

A total of 70 patients were included in the study: 34 metastatic and 36 non-metastatic. Patients with synchronous second primary cancers or prior chemotherapy treatment were excluded. Pathological factors (Ki-67 index, tumor grade, T and N stage), laboratory parameters (CA-15.3), and patient characteristics (age, weight, height, and body mass index [BMI]) were obtained from hospital records.

**Table 1:** Comparison of clinicopathological features between the CRELD2 positive group and the negative group in the study population

**Patients’ characteristics**		**Study population**		
		**CRELD2 negative group (Mean ± s.d.)**	**CRELD2 positive group (Mean ± s.d.)**	***P***
Age (years)		51.3 ± 11.3	53.8 ± 11.8	0.38
Weight (kg)		76.6 ± 14.2	73.4 ± 10	0.31
Height (m)		1.61 ± 0.06	1.61 ± 0.05	0.89
Body mass index (kg/m^2^)		29.3 ± 5	27.1 ± 6.3	0.1
CA-15.3 (U/mL)		16.2 ± 5.1	24.1 ± 4.4	0.36
Ki 67 (%)		50.7 ± 14	55.4 ± 11	0.84
**Patients’ characteristics**		**CRELD2 negative group** **(*n*)**	**CRELD2 positive group** **(*n*)**	***P***
Tumor grade	1	0 (0%)	1 (8.3%)	0.06
	2	23 (62.5%)	18 (75%)	
	3	19 (37.5%)	6 (16.7%)	
T stage	1	7 (16.2%)	8 (29.6%)	0.6
	2	27 (62.8%)	15 (55.6%)	
	3	6 (14%)	3 (11.1%)	
	4	3 (7%)	1 (3.7%)	
N stage	0	17 (35.5%)	9 (33.4%)	0.70
	1	14 (32.6%)	8 (29.6%)	
	2	5 (11.6%)	6 (22.2%)	
	3	7 (16.3%)	4 (14.8%)	

Characteristics of the study population. CRELD2: Cysteine-rich epidermal growth factor ligand domain 2.

Tissue samples for CRELD2 protein expression and immunohistochemical (IHC) staining were sourced from the pathology department’s archives. Overall survival (OS) was defined as the time from initial diagnosis to death from any cause. Progression-free survival (PFS) was defined as the time from initiation of first-line therapy in metastatic patients to radiological or clinical disease progression.

### CRELD2 IHC staining method

IHC staining was performed automatically using the Dako Omnis IHC device (Agilent, United States). The Dako DAB Detection Kit (Catalog No: K500711-2, United States) was utilized. Paraffin blocks were sectioned into 3-micron-thick slices using the LEICA RM2245 microtome and mounted onto positively charged slides. At least two distinct tissue samples were placed on each slide. The slides were baked at 70 ^∘^C in a Mega-Term E220P oven for 1 h before being processed on the Dako Omnis IHC device.

Antigen retrieval was performed using citrate buffer (pH 6.1). Antibody incubation was conducted with a 1:500 dilution of the concentrated Anti-CRELD2 antibody (Sigma ELISA Kit, Product No: HPA000603, Germany) for 25 min. Endogenous enzyme blocking was carried out for 3 min using PEROX, followed by enhancement with the secondary reagent EnVision FLEX + Rabbit Linker for 10 min. FLEX/HRP-labeled polymer was applied for 20 min, and Harris Hematoxylin was used for 5 min to achieve background staining.

The stained slides were examined by a pathologist using an Olympus BX46 microscope (Olympus Corporation, Japan). Tumor cell staining and the percentage of stained cells with cytoplasmic staining were assessed (Figure 1). Patients were categorized into two groups: CRELD2-positive and CRELD2-negative. Additionally, positive cases were further stratified based on the percentage of CRELD2 expression into three subgroups: 1%–10%, 11%–50%, and >50%.

### Statistical analysis

Data were analyzed using SPSS software (version 15.0, SPSS Inc., Chicago, IL, USA). Categorical variables between groups were compared using the chi-square test or Fisher’s exact test. Independent *t*-tests were used to compare continuous variables. Survival analysis was conducted using the Kaplan–Meier method, with comparisons made using the log-rank test. Risk factors for OS and PFS were assessed through a Cox regression model. Statistical significance was defined as *P* < 0.05.

## Results

Seventy patients were included in the study. Among them, 27 (38.6%) were CRELD2-positive, while 43 (61.4%) were CRELD2-negative. There was no significant difference in BMI between CRELD2-positive and CRELD2-negative patients (27.1 ± 6.3 kg/m^2^ vs 29.3 ± 5 kg/m^2^, *P* ═ 0.1). Similarly, no differences were observed in T stage, N stage, Ki-67 index, or tumor grade between the two groups (*P* > 0.05 for all; Table 1).

Of the 70 patients, 34 were metastatic, and 36 were non-metastatic. The median follow-up times were 46 months for the non-metastatic group and 38 months for the metastatic group. In the non-metastatic group, 12 patients (33.3%) were CRELD2-positive. Among these, nine (64%) had CRELD2 expression levels of 1%–10%, while three (36%) showed expression levels >50%.

In the metastatic group, 15 patients (44.1%) were CRELD2-positive. Of these, seven (46.8%) had CRELD2 expression levels of 1%–10%, four (26.6%) had 10%–50%, and four (26.6%) had >50%. The metastatic group included 15 patients (44.1%) with de novo metastases and 19 patients (55.9%) with recurrent disease. Among the de novo metastatic patients, nine (60%) were CRELD2-positive, while the remaining six were negative. In the recurrent group, six patients (31.5%) were CRELD2-positive, and 13 (68.5%) were negative.

Biopsy sites for the recurrent group included the liver (three patients), lungs (two patients), and lymph nodes (one patient). No significant difference was found in CRELD2 expression between primary and metastatic sites (*P* ═ 0.56).

In the non-metastatic group, no significant differences in clinicopathological findings were observed between the CRELD2-positive and CRELD2-negative groups (Table 2). In the survival analysis of non-metastatic patients, the five-year OS rate was 91.7% in the CRELD2-positive group and 91% in the CRELD2-negative group (*P* ═ 0.91).

**Table 2:** Comparison of clinicopathological features between the CRELD2 positive group and the negative group in the non-metastatic group

**Patients’ characteristics**		**Non-metastatic study population**		
		**CRELD2 negative group (Mean ± s.d.)**	**CRELD2 positive group (Mean ± s.d.)**	***P***
Age (years)		51.1 **±** 13.3	56.6 **±** 12.4	0.22
Weight (kg)		76.5 ± 17.0	75.2 ± 7.8	0.79
Height (m)		1.62 ± 0.07	1.63 ± 0.04	0.48
Body mass index (kg/m^2^)		29.1 ± 5.8	25.7 ± 8.5	0.17
CA-15.3 (U/mL)		18.4 ± 7.1	22.8 ± 5.4	0.26
Ki 67 (%)		51.1 ± 12	56.6 ± 13	0.73
**Patients’ characteristics**		**CRELD2 negative group** **(*n*)**	**CRELD2 positive group** **(*n*)**	***P***
Tumor grade	1	0 (0%)	1 (8.3%)	0.19
	2	15 (62.5%)	7 (75%)	
	3	9 (37.5%)	2 (16.7%)	
T stage	1	4 (16.7%)	4 (33.3%)	0.58
	2	16 (66.7%)	6 (50%)	
	3	3 (12.5%)	2 (16.7%)	
	4	1 (4.2%)	0 (0%)	
N stage	0	9 (37.5%)	5 (41.7%)	0.71
	1	8 (33.3%)	3 (25%)	
	2	3 (12.5%)	3 (82.5%)	
	3	4 (16.7%)	1 (8.3%)	

Characteristics of non-metastatic patients. CRELD2: Cysteine-rich epidermal growth factor ligand domain 2.

**Figure 2.**
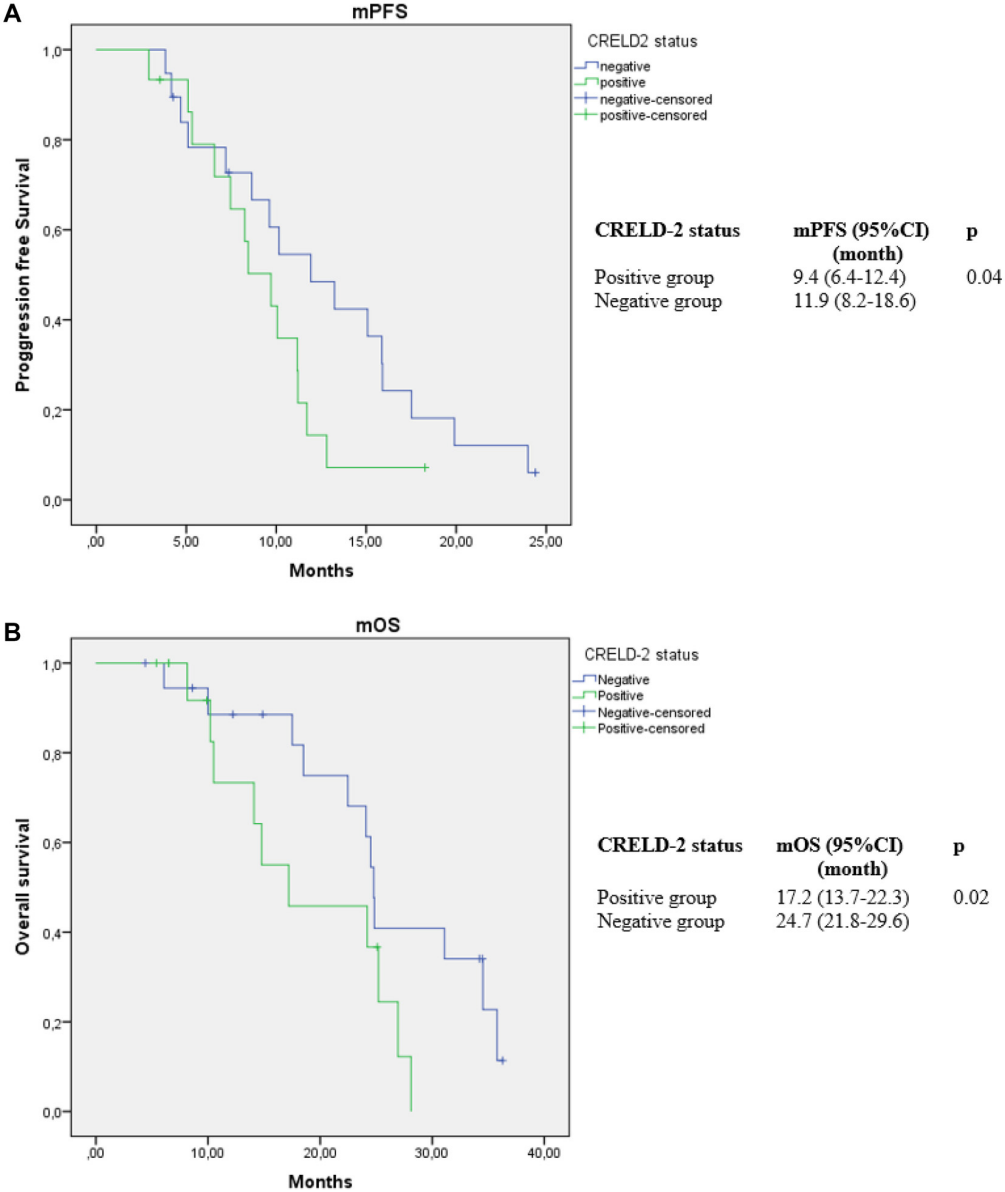
(A and B) Kaplan Meier curves for progression free survival (mPFS) and overall survival (mOS) according to CRELD2 positive and negative groups in the metastatic triple negative breast cancer. CRELD2: Cysteine-rich epidermal growth factor ligand domain 2; CI: Confidence interval.

Among metastatic patients, no statistically significant differences were found in clinical, laboratory, or pathological findings between the CRELD2-positive and CRELD2-negative groups (Table 3). However, the median progression-free survival (PFS) was 9.4 months (95% confidence interval [CI]: 6.4–12.4) in the CRELD2-positive group and 11.9 months (95% CI: 8.2–18.6) in the CRELD2-negative group (*P* ═ 0.04). The median OS was 17.2 months (95% CI: 13.7–22.3) in the CRELD2-positive group compared to 24.7 months (95% CI: 21.8–29.6) in the CRELD2-negative group (*P* ═ 0.02) (Figure 2).

**Table 3:** Comparison of clinicopathological features between the CRELD2 positive group and the negative group in the metastatic group

**Patients’ characteristics**		**Metastatic study population**		
		**CRELD2 negative group (Mean ± s.d.)**	**CRELD2 positive group (Mean ± s.d.)**	***P***
Age (years)		51.6 ± 10.1	51.6 ± 10.4	0.98
Weight (kg)		76.6 ± 9.9	72 ± 12.1	0.22
Height (m)		1.61 ± 0.06	1.59 ± 0.05	0.48
Body mass index (kg/m^2^)		29.5 ± 3.8	28.1 ± 3.9	0.3
CA-15.3 (U/mL)		44.8 ± 4.5	48.1 ± 5.7	0.28
Ki 67 (%)		57.3 ± 24	57.9 ± 27	0.95
**Patients’ characteristics**		**CRELD2 negative group** **(*n*)**	**CRELD2 positive group** **(*n*)**	***P***
Tumor grade	2	8 (42.1%)	11 (73.3%)	0.07
	3	11 (57.9%)	4 (26.7%)	
T stage	1	3 (15.8%)	4 (26.6%)	0.74
	2	11 (57.9%)	9 (60%)	
	3	3 (15.8%)	1 (6.7%)	
	4	2 (10.5%)	1 (6.7%)	
N stage	0	8 (42.1%)	4 (26.7%)	0.8
	1	6 (31.6%)	5 (33.3%)	
	2	2 (10.5%)	3 (20%)	
	3	3 (15.8%)	3 (20%)	
Surgery for primary	Yes	17 (89.5%)	11 (73.3%)	0.37
	No	2 (10.5%)	4 (26.7%)	
Receiving adjuvant chemotherapy	Yes	12 (63.2%)	9 (60%)	084
	No	9 (36.8%)	6 (40%)	
Response to treatment	Partial	9 (47.3%)	7 (46.7%)	0.43
	Stable	6 (31.6%)	7 (46.7%)	
	Progression	4 (21.1%)	1 (6.6%)	

Characteristics of metastatic patients. CRELD2: Cysteine-rich epidermal growth factor ligand domain 2.

**Table 4:** Univariate and multivariate analysis for prognostic factors on OS and PFS in metastatic triple negative breast cancer

**OS risk factors**		**Univariate analysis**			**Multivariate analysis**		
		**HR**	**95% CI**	***P***	**HR**	**95% CI**	***P***
CRELD2	Negative vs positive	0.62	0.25–0.96	0.03	0.82	0.33–0.96	0.01
CRELD2 percentage (%)	Negative	Reference		0.23	–		–
	1%–10%	1.16	0.364–3.745	0.79	–	–	–
	11%–50%	1.31	0.279–6.298	0.72	–	–	–
	>50%	3.58	0.94–12.28	0.43	–	–	–
Age (years)		1.05	1.008–1.10	0.02	1.06	0.95–1.20	0.27
Body mass index (kg/m^2^)		1.07	0.93–1.24	0.28	1.12	0.89–1.41	0.32
Denovo metastatic	No vs Yes	0.67	0.28–1.60	0.37	–	–	–
Surgery for primary	Yes vs No	0.27	0.089–0.87	0.028	20.5	1.93–218.3	0.012
Ki-67 (%)		0.99	0.97–1.08	0.32	0.95	0.91–0.99	0.09
Tumor grade	3 vs 2	1.36	0.53–3.48	0.51	–	–	–
T stage	1	Reference		0.57	Reference		0.77
	2	1.19	0.25–5.5	0.81	0.061	0.06–5.64	0.66
	3	2.89	0.47–17.5	0.24	8.7	0.22–34.6	0.24
	4	1.40	0.18–10.5	0.74	0.05	0.001–2.09	0.11
N stage	0	Reference		0.21	Reference		0.04
	1	1.47	0.42–506	0.53	2.9	0.16–52.3	0.46
	2	3.2	0.78–12.99	0.1	21.48	1.37–33.3	0.029
	3	3.1	0.85–11.41	0.08	34.21	1.05–110.9	0.04
**PFS risk factors**		**Univariate analysis**			**Multivariate analysis**		
		**HR**	**95% CI**	***P***	**HR**	**95% CI**	***P***
CRELD2	Negative vs positive	0.71	0.34–0.95	0.04	0.50	0.38–0.96	0.02
CRELD2 percentage (%)	Negative	Reference		0.71	–		–
	1%–10%	1.45	0.55–3.83	0.44	–	–	–
	11%–50%	0.99	0.28–3.46	0.98	–	–	–
	>50%	1.75	0.57–5.37	0.32	–	–	–
Age (years)		1.01	0.98–1.05	0.34	0.96	0.90–1.03	0.29
BMI (kg/m^2^)		1.05	0.93–1.19	0.39	1.08	0.92–1.28	0.31
Denovo metastatic	No vs Yes	0.91	0.43–1.91	0.80	–	–	–
Surgery for primary	Yes vs No	2.22	0.87–5.64	0.092	4.3	0.95–20.1	0.057
Ki-67 (%)		0.99	0.97–1.01	0.6	1.01	0.97–1.024	0.9
Tumor grade	3 vs 2	1.67	0.74–3.79	0.21	–	–	–
T stage	1	Reference		0.53	Reference		0.07
	2	2	0.66–6.03	0.2	7.1	1.13–44.8	0.036
	3	1.4	0.34–5.69	0.63	1.89	0.17–21	0.6
	4	3	0.5–17.95	0.22	1.20	0.055–26.05	0.9
N stage	0	Reference		0.15	Reference		0.1
	1	1.34	0.41–3.49	0.55	0.86	0.22–3.30	0.83
	2	3.32	0.92–11.9	0.065	2.86	0.55–14.7	0.2
	3	2.97	0.97–9.1	0.057	5.3	1.14–24.6	0.033

Risk factors for metastatic patients. OS: Overall survival; PFS: Progression free survival; BMI: Body mass index; CI: Confidence interval; CRELD2: Cysteine-rich epidermal growth factor ligand domain 2.

Survival analysis was also performed based on the percentage of CRELD2 expression. For patients with 1%–10% CRELD2 expression, the median OS was 25.2 months (95% CI: not estimated [NE]) and the median PFS was 10.4 months (95% CI: 7.4–13.4). In patients with CRELD2 expression between 11% and 50%, the median OS was 21.4 months (95% CI: NE) and the median PFS was 10 months (95% CI: 2.48–17.62). For patients with > 50% CRELD2 expression, the median OS was 13.9 months (95% CI: 7.03–24.8; *P* ═ 0.18), and the median PFS was 6.5 months (95% CI: 2.12–11.01; *P* ═ 0.64).

In the metastatic patient group, CRELD2 status, CRELD2 expression percentage, body mass index, de novo metastasis, surgery for the primary tumor, Ki-67 level, tumor grade, T stage, and N stage were evaluated as risk factors for OS using univariate Cox regression analysis (Table 4). In the multivariate analysis, CRELD2 status (negative vs positive) (hazard ratio [HR]: 0.50, 95% CI: 0.38–0.96, *P* ═ 0.02), surgery for the primary tumor, and N2-3 stages were identified as significant risk factors for OS. Additionally, CRELD2 status (negative vs positive) (HR: 0.82, 95% CI: 0.33–0.96, *P* ═ 0.01), T2 stage, and N3 stage were determined to be risk factors for progression-free survival (PFS) in patients with metastatic TNBC (Table 4).

## Discussion

This is the first clinical study to evaluate the effect of CRELD2 on survival and its prognostic significance in TNBC patients. In our analysis, we found that CRELD2 positivity and expression did not influence survival outcomes in patients with non-metastatic TNBC.

One of the most intriguing aspects of CRELD2’s role in TNBC is its involvement in the ER stress response. TNBC tumors are highly heterogeneous and aggressive, often characterized by hypoxic and nutrient-deprived microenvironments. Under these conditions, the unfolded protein response is activated to mitigate ER stress and promote tumor cell survival. As an ER-resident protein, CRELD2 may play a critical role in this adaptive response, potentially driving tumor progression and therapy resistance [15, 19].

A recent study identified CRELD2 as a driver of tumor progression [13]. This study [13] also found a significant association between high CRELD2 expression and decreased survival in breast cancer patients, with particular relevance to the triple-negative subtype. Moreover, additional studies have highlighted key factors in TNBC pathophysiology. For example, Processing of Precursors 1 has been shown to promote TNBC proliferation by degrading CDKN1A mRNA [20], while LYPLAL1-DT exhibits anti-oncogenic effects in TNBC [21].

Data from the Human Protein Atlas database further underscore the complex prognostic role of CRELD2, identifying it as an unfavorable marker in kidney cancer but a favorable marker in endometrial cancer [22, 23]. CRELD2 may also mediate tumor angiogenesis [24] and serve as a novel androgen receptor target in prostate cancer [25].

In this study, CRELD2 positivity was associated with shorter OS and PFS in patients with metastatic TNBC. Notably, CRELD2 positivity emerged as a predictor of worse OS and PFS specifically in metastatic TNBC.

TNBC is unresponsive to endocrine or molecular-targeted therapies [26], leaving limited treatment options. Ongoing research aims to identify additional biomarkers and targeted therapies to improve clinical outcomes [27]. A recent study discovered that CRELD2-mediated disruption of tumor–stroma crosstalk presents a potential therapeutic target, emphasizing the importance of CRELD2 in patients with metastatic TNBC.

Patients with TNBC generally have poorer survival rates compared to those with other breast cancer subtypes, with a mortality rate of 40% within the first five years post-diagnosis [28, 29]. Prognostic factors for TNBC typically include lymph node status, tumor size, age, BMI, menopausal status, lymphatic/vascular invasion, and histologic grade [30–33]. Additionally, the number of CD4+ and CD8+ T cells, the CD4/CD8 ratio at the tumor site, and CD30 expression levels are considered potential indicators for prognosis and therapeutic intervention in invasive breast carcinoma [34].

In the current study, lack of surgery for the primary tumor and extensive lymph node involvement were identified as poor prognostic factors for OS. Similarly, large tumor size and high lymph node involvement were linked to worse PFS, aligning with findings reported in the literature.

This study had several limitations. First, it was retrospective in nature. Second, the sample size was relatively small.

## Conclusion

This is the first clinical study to evaluate the effect of CRELD2 on survival and its potential as a prognostic marker in patients with TNBC. These findings should be validated in prospective studies with a larger sample size.

## Data Availability

The data presented in this study can be obtained from the corresponding author.
